# Modelling the evolution of cognitive styles

**DOI:** 10.1186/s12862-019-1565-2

**Published:** 2019-12-27

**Authors:** Jannis Liedtke, Lutz Fromhage

**Affiliations:** Department of Biological and Environmental Science, University of Jyvaskyla, Box 35, 40014 Jyvaskyla, PO Finland

**Keywords:** Coping style, Behaviour syndromes, Learning, Cognition, Exploration, Animal intelligence

## Abstract

**Background:**

Individuals consistently differ in behaviour, exhibiting so-called personalities. In many species, individuals differ also in their cognitive abilities. When personalities and cognitive abilities occur in distinct combinations, they can be described as ‘cognitive styles’. Both empirical and theoretical investigations produced contradicting or mixed results regarding the complex interplay between cognitive styles and environmental conditions.

**Results:**

Here we use individual-based simulations to show that, under just slightly different environmental conditions, different cognitive styles exist and under a variety of conditions, can also co-exist. Co-existences are based on individual specialization on different resources, or, more generally speaking, on individuals adopting different niches or microhabitats.

**Conclusions:**

The results presented here suggest that in many species, individuals of the same population may adopt different cognitive styles. Thereby the present study may help to explain the variety of styles described in previous studies and why different, sometimes contradicting, results have been found under similar conditions.

## Background

Joining studies of individual differences in cognition and of animal personalities leads to the field of “cognitive styles”. The concept of cognitive styles describes how individuals consistently differ in how they use their cognitive capacities in combination with consistent inter-individual differences in behaviours such as exploration, boldness or aggressiveness (reviewed in [1–5]). Empirical data support the existence of different cognitive styles in nature (e.g. [6–8]). Furthermore, the existence of animal personality in virtually all species tested (e.g. [9–11]), combined with the fast-growing body of evidence of individual differences in cognitive abilities within species (reviewed in [5]) let it seem likely that different cognitive styles can be found in a vast variety of species and that this constitutes an important ecological and evolutionary aspect.

Interestingly, empirical studies often show opposing findings [reviewed in 8] and based on these and theoretical considerations different and contradicting predictions about cognitive styles have been formulated (see e.g. [1–3]). Probably the most influential of these, the proactive-reactive framework, states that „proactive “individuals tend to be bold and explorative, forming behavioural routines quickly, but having trouble to incorporate new information about the environment [2]. The latter may limit the performance of this behavioural type in many cognitive tasks. On the opposite end of this continuum are the so-called „reactive “individuals, which tend to be shy and less explorative but more sensitive towards environmental cues and opportunities in their surroundings. It has been hypothesized that these individuals should be better at dealing with some cognitive challenges, especially when the tasks require to reverse previously formed associations [2]. And indeed, experimental studies have found supporting evidence for these behaviour/cognitive types in some species [reviewed in e.g. 2, 8]. However, other studies found different combinations of behavioural and cognitive characteristics, contradicting the proposed behavioural and cognitive types of the „proactive-reactive “framework. For example, in some fish [12], bird [13, 14], and mammal [15] species, bolder or more explorative individuals were generally better at cognitive tasks than shyer individuals. Yet other studies could find only mixed, weak, or even no correlation between cognitive performance and exploration or activity level (e.g. [16–18]).

At a first glance, the variation in superficially opposing findings and predictions may come as a surprise. However, nature is complex and often these opposing finding derive from different study systems with different ecological context. Therefore, while some differences in these results may be explained by methodological design (compare [19]), many of the demonstrated differences in previous studies may be ecologically meaningful and reflect differences in the evolution and development of cognitive styles. It has been shown that the expression of traits underlying cognitive styles can crucially depend on environmental conditions (personality traits (e.g. [20–22]); cognition (e.g. [23–25]); brain morphology (e.g. [26–28]).

In particular, predation pressure is regarded as a major environmental factor which may strongly influence the development of consistent inter-individual differences in behaviour (e.g. [29–31], but see [32]). Based on the above-stated findings and considerations, it seems that many different cognitive styles can emerge depending on the precise ecological circumstances in which individuals live. To complement this view, the primary aim of this study is to investigate if different cognitive styles can also emerge under similar environmental conditions and whether they may coexist in the same environment.

Furthermore, similar to the above-mentioned contrasting predictions about which cognitive styles should exist, opposing suggestions have been formulated as to whether behavioural traits influence the evolution or development of cognitive abilities or vice versa. On the one hand, behaviour may shape the development of cognitive abilities [2]. On the other hand, it has been suggested that cognition may in turn influence personality (responsiveness in particular) [2, 3]. Both possibilities seem plausible both at ontological and evolutionary timescales and may feed back on each other.

Using individual-based simulations, we want to investigate i) whether different cognitive styles can evolve under different environmental conditions, thereby helping to explain apparently contradicting evidence from experimental and theoretical studies, ii) whether even within the same environment, different cognitive styles can coexist, which may help to explain the existence of large differences in cognitive abilities within a species, and iii) whether behavioural and cognitive traits can influence each other’s evolution. While in nature a huge variety of factors will influence these issues, we concentrate here on two traits of individuals (namely, exploration tendency and learning ability) and two features of the environment (namely, complexity in terms of different resource types, and predation pressure). Taking these four variables, we investigate the effect of environmental conditions on the evolution of learning skills and exploration tendency in individuals of the same population. While the presented simulations are based on genetic adaptations, the general conclusions should also hold for developmentally plastic systems, which should likewise produce phenotypes that are adapted to local conditions. Our results can help explain apparently contradicting findings of previous studies and outline complex interactions between individual traits and environmental conditions in regard to the evolution of cognitive styles.

## Methods

The models presented here are an extension of a model used in previous work [33]. We implemented populations of *N*_*Individuals*_ individuals in which three traits can evolve independently: learning ability *L,* exploration tendency *E,* and selectiveness *S*. Both *L* and *E* are continuous traits and can take values between 0 and 1. *S* is binary and can be either 0 or 1. Simulations are run for *N*_*Generations*_ discrete generations (= seasons). At the end of each season, we let individuals reproduce asexually in relation to their fitness. Fitness is determined by the amount and value of resources an individual collected throughout its lifetime. Each season has *T* days, which sets the maximum lifespan of individuals. Each day consists of *N*_*Steps*_ steps through which each individual proceeds. In the beginning of each day, the order of individuals is randomized to ensure equal chances.

### Environment

The environment consists of a number of *N*_*Sites*_ sites, each of which can either contain one of two types of resources (R1 or R2) or can be empty. Resources are randomly distributed at the beginning of the season, such that *P*_*Ri*_ is the proportion of sites filled with resource type *R*_*i*_. Resources are defined by their value *V*_*Ri*_*,* their handling-time *H*_*Ri*_*,* and their detectability *D*_*Ri*_*,* i.e. how difficult they are to find.

### Predation

Predation is implemented by introducing three different predator types (P1, P2, and P3), which are defined by their baseline probability of being encountered (*P*_*p*_) and their lethality *λ*_*P*_*,* i.e. how likely an individual will die when encountering this predator type. Whenever an individual moves in order to explore its environment, it is vulnerable to predation. We calculate the probability of a predator attack from a binomial distribution as:

1 $$ {P}_{attack}=1-\left[\left(1-{P}_{P1}\right)\ast \left(1-{P}_{P2}\right)\ast \left(1-{P}_{P3}\right)\ast \left(1-{E}_k\right)\ast 0.1\right] $$

Here, *E*_*k*_ is the focal individual’s exploration tendency. Thus, the more explorative an individual is, i.e. the more it moves around, the more likely a predator attack becomes. When an attack happens, the type of predator is sampled according to the relative probabilities of the predator types (P_P1_, P_P2_, P_P3_). The individual under attack survives with probability:

2 $$ {P}_{survival}=1-{\lambda}_P $$

### Actions

During each time step, an individual can perform one of the following actions: rest (and hide), explore (searching for resources), handle resources, or escape a predator. In the beginning of each day, or in any time step after an action has been completed, it is determined whether an individual will move in the current time step. If the individuals’ *cumulative exploration tendency* (*C*) is above a randomly drawn threshold (between 0 and 1) it will move; otherwise it will rest. *Cumulative exploration tendency* means that each time an individual rests its *C* will increase by the value of *E*_*k*_. For example, if the focal individual has an *E* = 0.3 and has rested for the previous two time steps, its *C* in the current time step becomes = 0.9. Thus, it will move with 90% probability. Consequently, individuals with *E* > = 0.5 never rest more than once in a row. *E* is genetically encoded by a single locus, whose initial allelic values are randomly sampled from a uniform distribution between 0 and 1.

If an individual moves it will visit a randomly chosen site. Here it may encounter a predator with the probability given in Eq.1 and survive its attack with the probability given in Eq. 2. If it survives, it has the possibility to learn anti-predation behaviour (see below) after which the time step is over. If it dies, the individual will not participate in any further actions. If a moving individual is not attacked, it can explore the randomly chosen site and search for resources. If it enters a site containing a resource, it finds the resource with probability

3 $$ {P}_F=1-{E}_k\ast \left(1-{D}_{Ri}\right) $$

Individuals can then start handling the resource and, depending on the handling time of this resource type, obtain its value. If the handling time is larger than 1, the individual can continue to reduce the initial handling time by 1 unit in each following time step, until the residual handling time reaches 0 and the resource value is gained. When only one time step is left in the current day, the individual needs to stop handling the resource and return to its hiding place without obtaining the reward. When a resource was successfully exploited, the site it was found in was emptied and not refilled. Thus, any exploitation of a resource reduces the likelihood of finding a resource in subsequent exploration attempts for all individuals until the end of the season.

### Learning

We implement resource-learning as a reduction of handling time due to having experience with a given resource type. Each time an individual ends handling a resource type with larger handling times than a given minimum (*Hmin* = 3 in all presented cases), the handling time for this individual and this resource type is updated as:

4 $$ {h}_i=\max \left[3,{h}_i-L\ast t/{h}_{\left(i, Initial\right)}\right] $$

Here, *L* is the focal individual’s learning speed; *t* is the number of time steps spent handling the resource item; *h*_*i,Initial*_ is the initial handling time for resource type *R*_*i*_ at the beginning of the current encounter; and *t*/*h*_*(i,Initial)*_ is the proportion of the learning episode that was completed. The maximization function max[.] ensures that handling times cannot drop below 3 (i.e. *Hmin*). *L* is genetically encoded by a single locus, whose initial allelic values are randomly sampled from a uniform distribution between 0 and 1.

Similarly to resource learning, lethality of a predator type can be reduced through learning each time an individual survives an attack. After an unsuccessful attack by a specific predator, the current lethality of this predator type is updated for the focal individual as:

5 $$ {\lambda}_P={\lambda}_P-{\lambda}_P\ast L\ast \beta $$

Here, *L* is the focal individual’s learning speed; *λ*_*P*_ is the current lethality for this predator type, which is identical for all individuals at the beginning of the season (i.e. before any learning took place) and *ß* is a parameter defining the general speed of predation-learning. The lethality of predators could not be reduced lower than 1/10 of their original value (at the beginning of the season before any learning took place). Thus, predators always have a minimum lethality no matter how often an individual has survived an attack of that predator type.

### Selectiveness

We implemented individuals as being either *selective* or *non-selective* foragers. *Selective* individuals handle only resources whose handling time they can complete by the end of the day. Resources with longer handling times were rejected immediately and individuals can move to a new site in the next time step. *Non-selective* individuals handle any resources they find. This can lead to handling being interrupted prematurely at the end of the day, yielding no immediate reward. Yet, such incomplete handling of resources still provides an opportunity for learning. Therefore, *non-selective* individuals can eventually learn to collect resources whose initial handling times exceed a day’s length. *Selectiveness* is genetically implemented by one locus with two alleles, determining individuals to be either *selective* (*S* = 1) or *non-selective* (*S* = 0). The initial allelic values are randomly sampled with equal probability.

### Reproduction

We assumed an ‘income breeder’ system where all individuals, independently of their survival until the end of the season, produced offspring in relation to the total amount of the value of collected resources throughout their lifetime. Reproductive success is calculated as:

6 $$ F={V}_{Total}\ast \left(1-\alpha \ast L\right) $$

where *V*_*Total*_ is the total value of collected resources, *L* is the individual’s learning speed, and *α* is a cost coefficient that specifies the cost of learning. No costs of exploration (*E*) are explicitly included in this calculation, as they are implicit in the risk of overlooking resources and attracting predators. The next generation is recruited by randomly sampling offspring from the present generation, using *F* as the independent sampling probabilities.

### Mutation

All three traits, *L*, *E*, and *S* were independently subject to mutation. Mutation probability is set to *q* = 0.1 for each trait. For the continuous traits *L* and *E*, new trait values were chosen randomly from a normal distribution, with a mean of the parental trait value and a SD of 0.1. For the binary trait *S,* a mutation event would change the value from one state to the other (i.e. from 1 to 0 and vice versa).

In order to investigate whether behaviour influences the evolution of cognitive abilities or vice versa we ran an additional set of simulations where, for the first 100 generations, either the trait *L* or *E* was set to an arbitrary chosen and fixed value for all individuals. Only after these 100 generations, we allowed mutations for the constrained trait as well. In this way we could observe how much the other trait would alter its value after the constrained trait was allowed to change. We ran simulations with either high and low starting values for the initially constrained trait. As before, mutation probability was set to *q* = 0.1 for the unconstrained trait and for the constrained trait after 100 generations. New trait values were chosen randomly from a normal distribution, with a mean of the parental trait value and a heightened SD of 0.3 to increase the speed of adaptation.

## Results

**Fig. 1:**
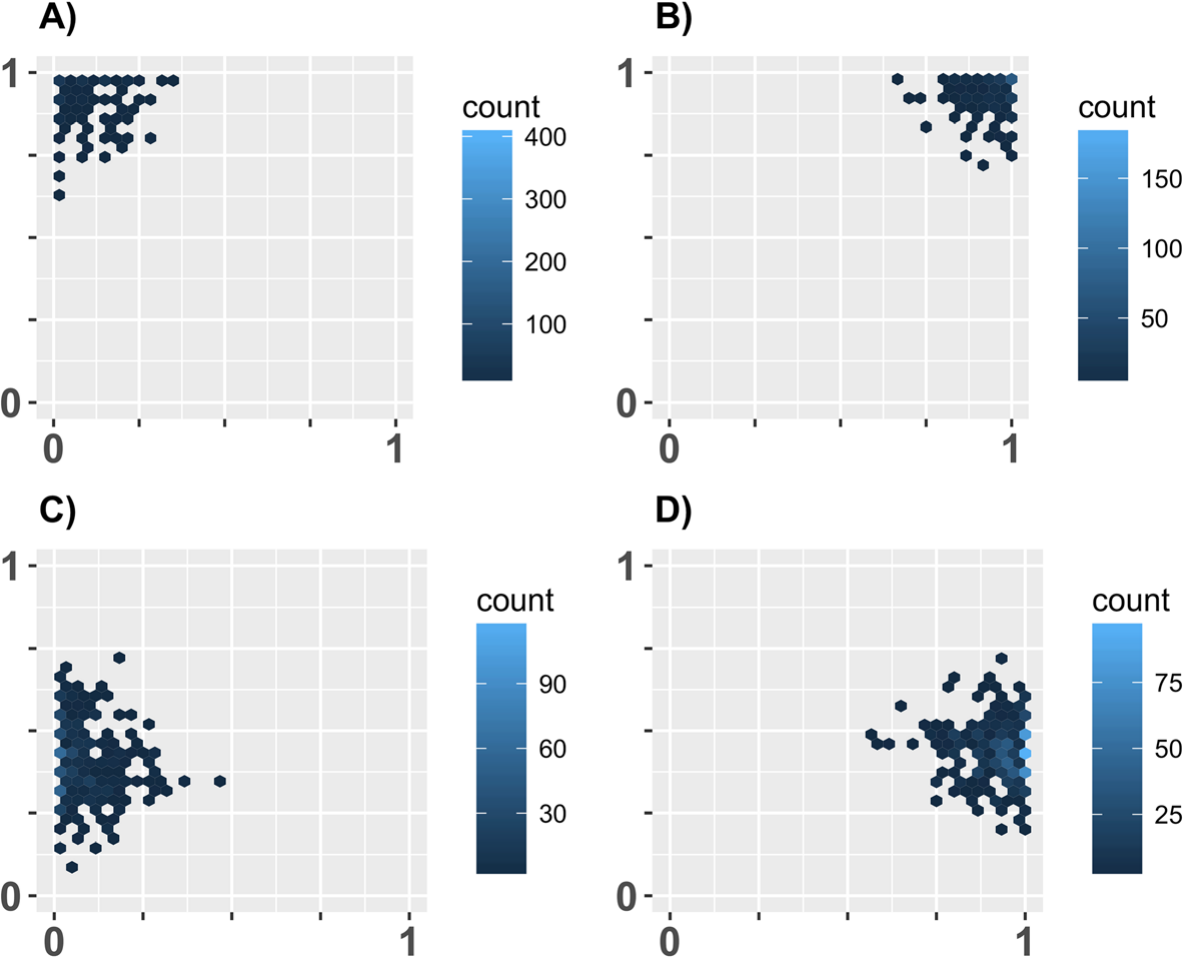
Different cognitive styles are adaptive under slightly different conditions. Each panel shows the result of one simulation as an example from 10 replicate runs. All replicates produced qualitatively similar results. Each simulation was run with *N* = 1000, *G* = 500 and without predation. The only differences in parameter setting between panels were in resource detectability (*D*_*Ri*_) and season length (*T*). Only in case of **b**), an increase in resource abundance of R2 was needed to ensure that an alternative strategy did not coexist with the shown cognitive style. Settings: **a**) *D*_*R1*_ = 0.9 and *D*_*R2*_ = 0.9; *T* = 15; B) *D*_*R1*_ = 0.9 and *D*_*R2*_ = 0.9; *T* = 60; **c**) *D*_*R1*_ = 0.0 and *D*_*R2*_ = 0.9; *T* = 10; **d**) *D*_*R1*_ = 0.0 and *D*_*R2*_ = 0.0; *T* = 120

**Fig. 2:**
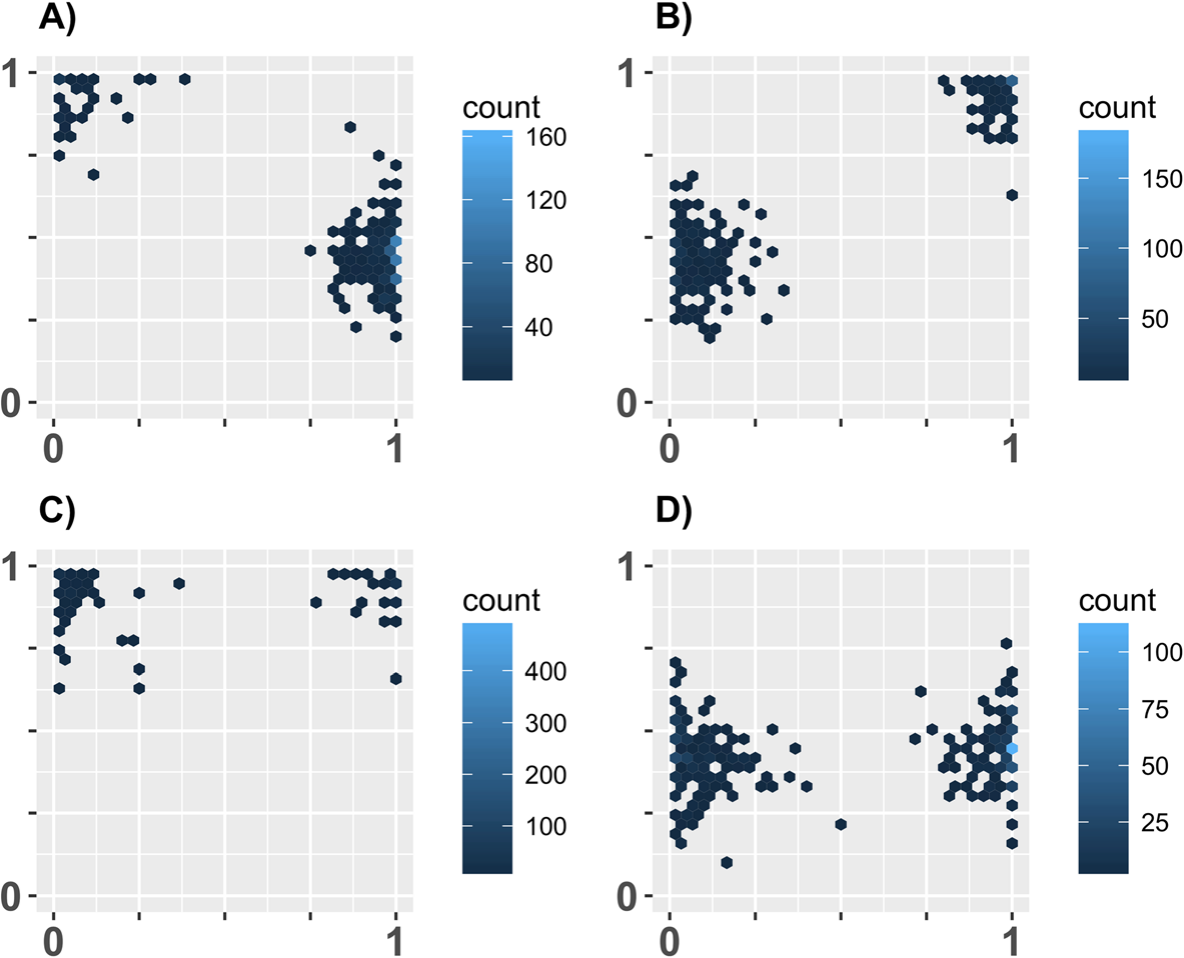
Co-existence of different cognitive styles within the same environment. Each panel shows the result of one simulation as an example from 10 replicate runs. All replicates produced qualitatively similar results. Each simulation was run with *N* = 1000, *G* = 500 and without predation. The only differences in parameter setting between panels were in resource detectability (*D*_*Ri*_) and season length (*T*). Settings: **a**) *D*_*R1*_ = 0.9 and *D*_*R2*_ = 0.0; *T* = 60; **b**) *D*_*R1*_ = 0.0 and *D*_*R2*_ = 0.9; *T* = 90; **c**) *D*_*R1*_ = 0.9 and *D*_*R2*_ = 0.9; *T* = 20; **d**) *D*_*R1*_ = 0.0 and *D*_*R2*_ = 0.0; *T* = 45

We heuristically explored the parameter space for conditions where we could find the existence of different cognitive styles with changing (for simplicity) as few parameters as possible. For the main findings we therefore changed the value of only three parameters unless stated otherwise. We found circumstances under which different combinations of the two individual traits *L* and *E* predominated in the population (see Fig. 1). We also found various cases of two different cognitive styles coexisting within the same population (see Fig. 2). The values of only two parameters needed to be changed in order to find these results. One is the detectability (*D*_*Ri*_), which was either low (0) or high (0.9) for either resource type (R1 or R2). The other parameter was the length of season (*T*; i.e. maximal lifespan of individuals). Only in order to get a pure high *E* high *L* cognitive style (Fig. 1 b) we needed to increase the abundance of the high value resource so that an alternative style, which exploited low value resources, was not adaptive even for a small portion of the population. Predation pressure (i.e. how likely an attack occurred and how lethal this attack was) was not needed to obtain these results. Nevertheless, this factor had a strong influence (see below).

**Fig. 3:**
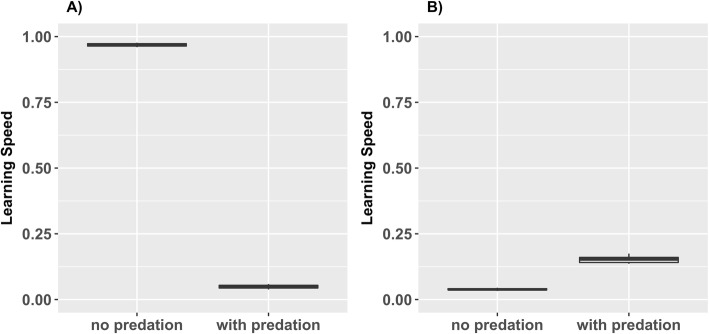
Effects of predation. **a**) Predation can prevent emergence of fast-learning cognitive styles. Under conditions without predation, trait *L* evolved to be high in order to exploit resource R2. With predation, *L* evolved to be low, because individuals could not learn to handle resource R2 anymore due to the decreased lifespans caused by predation. Besides predation pressure, the enviroments are identical (*D*_*R1*_ = 0.0 and *D*_*R2*_ = 0.0; *T* = 90). Boxplots are based on 10 replicate simulations with *N* = 1000 and *G* = 500. B) Predation pressure could also lead to the evolution of elevated *L*. Please note that we set the handling time for resource R2 very high (*H*_*R2*_ = 500), so that it could not be reduced within the lifetime of individuals. Thus, in this example, increased *L* was solely beneficial in regard to antipredation behaviour. Therefore, in this example, high *L* was induced by predation pressure. Boxplots are based on 10 replicates simulations with *N* = 1000 and *G* = 500

As expected, we found no investment into learning (low *L*) whenever there was nothing to learn, i.e. handling time of resources were low and predators were absent. Additionally, this could occur whenever individuals could not learn fast enough because the season length (lifespan) was too short or predation pressure was so high that individuals were killed before they could learn sufficiently. Thus, in this way, predation could prevent the existence of ‘fast learning’ styles (see Fig. 3a). On the other hand, predation pressure could also lead to the evolution of high *L* in an otherwise „non-learning” environment (i.e. in an environment with only resources with low handling times or when exploiting resources with high handling times was not worth learning for). If predation pressure was not too severe, individuals could benefit from investing into learning abilities in order to reduce predation pressure and increase their expected lifespan, thereby increasing the overall income of resources (see Fig. 3b). Furthermore, predation could also hinder the existence of high exploration tendencies (high *E*) because the faster one explores, the more likely predators were attracted (see Additional file 1: Figure S1).

Exploration tendency also depends strongly on how easily resources were detected. When resources are conspicuous, individuals can find them even when exploring fast; hence high *E* becomes adaptive. However, whenever resources are hard to find (i.e. *D*_*Ri*_ is low) low *E* can yield higher payoffs as it ensures that resources are not overlooked. Note that, since individuals need to explore in order to find something at all, minimum *E* (> 0) are to be expected. In our simulations without predation, the optimal exploration tendency was around ~ 0.4. Due to the *cumulating exploration tendency* (*C*) this value of *E* ensures that individuals will most likely explore at least every second time step, while keeping the risk of overlooking resources moderately low. However, high exploration may be needed when life is very short, so that to ensure finding any resources at all, individuals need to explore each time step – regardless of the risk of predation and of overlooking resources.

We found coexistence of cognitive styles when individuals specialize in exploiting one of the two resource types (Fig. 2). In the results presented here, R1 was always a low-valued resource (*V*_*R1*_ = 1) which did not necessitate any learning, while R2 always had a high handling time (*H*_*R2*_ = 15), which could be reduced through learning and was higher-valued (*V*_*R2*_ = 15). Coexistence under these conditions can occur, for example, when the high-valued resource (R2) has a long handling time that necessitates learning, while being relatively rare. Some individuals may then invest into high *L*, whereas others will instead explore fast and exploit less-valued but more abundant resources (R1). Due to negative frequency-dependence this pattern can also occur the other way around when we tweak the parameters a little, so as to make the more valuable R2 resource relatively easier to find. Most individuals then invest in high *L* and exploit the more valuable and now easier-to-find R2 resources. Some individuals, however, will avoid competition and specialize on less-valued R1 resources even if they are hard to find. But since most other conspecifics will not exploit them (as they mostly overlook them while quickly exploring for easy-to-find R2) the less common ‘slow explorer’ can find relatively many R1 and thereby gain a similar payoff as fast-exploring and fast-learning individuals searching for R2 (Fig. 2).

Qualitatively similar results can also be obtained with other ratios between the values of low- versus high-valued resources, provided the parameters “lifespan” or “events per day” are adjusted accordingly. The general principle is that there must be enough time for the type specializing on the more valuable (but harder to learn) resource to recoup its initial investment in learning. This recouping may take place either through a few highly profitable events, or through a large number of much less profitable events.

Predation influenced co-existence of two cognitive styles as well. Within a wide range of parameter space, predation can hinder the co-existence, by making fast exploration less beneficial (Additional file 1: Figure S2a-b). Moreover, predation can also make slow learning less beneficial (Additional file 1: Figure S2 c), as slow learners are not able to learn sufficiently to reduce lethality of predators. Or, under conditions where even fast learning will not reduce predation sufficiently, fast learning styles are prevented (Additional file 1: Figure S2 d). Yet, within a narrow parameter space, predation can also induce co-existence (Additional file 1: Figure S3) by reducing the payoffs of a fast learning style, making a slow learning strategy competitive. (Although in one out of ten simulation runs, the co-existence of two styles collapsed due to the extinction of the fast learning strategy. This was likely caused by a combination of stochastic events and high predation pressure.)

Coexistence can also occur when individuals of both cognitive styles show the same exploration tendency (*E*) (Fig. 2c and d). This can occur when both types of resources (R1 and R2) are easy to find and thus select for fast exploring (Fig. 2c). Some individuals may then specialize on more abundant R1, with low handling times but lower value. Other individuals invest in higher *L* and exploit R2, which need to be higher-valued. Thus, a fast-exploring and slow-learning cognitive style can occur alongside a fast-exploring and fast-learning style within the same environment. Similarly, when both resource types are hard to find, two cognitive styles with low *E* can coexist if some individuals specialize on low-valued but easy-to-exploit resources (R1) and others on high-valued but hard-to-exploit resources (R2) (Fig. 2d). These coexistences, which arise due to negative-frequency dependence, can be found in a moderately wide range of parameters space.

We also expected to find the coexistence of different cognitive styles with the same learning strategy (*L*). However, we could not find any parameter space in which either low learning could exist in combination with both high and low exploration, or in which fast learning strategy could exist in combination with both high and low exploration. Even though in our present model we could not find evidence for this, that does not mean that these styles could not coexist in any model or environment.

Finally, in the simulations where either of the traits was allowed to evolve only after an initial period of being fixed, we observed that the respective other trait changed its value in response to changes of the initially fixed trait. This demonstrates how behavioural traits may influence the evolution of cognitive abilities (Additional file 1: Figure S4 a and b) and vice versa (Additional file 1: Figure S4 c and d).

## Discussion

We found that combinations of the environmental factors „resource composition “and „predation “can select for a variety of cognitive styles. Depending on the value of these factors, our results are in line with the overall predictions of the proactive-reactive framework [2]: under certain circumstances, proactive (reactive) individuals invest less (more) in learning abilities. However, under just slightly different environmental conditions, the patterns are reversed, thereby being consistent with findings which oppose the predictions of the proactive-reactive framework. Showing how sensitive the occurrence of cognitive styles toward environmental circumstances can be in theory provides context for interpreting the vast variation that has been empirically observed. This responsiveness is consistent with Niemelä and Dingemanse’s [34] view that non-linear relationships such as thresholds and interactions are common in animal personalities.

How can we explain the specific patterns observed in our simulations? For example, in dangerous environments, in which resources are easy to exploit and thus do not necessitate any learning, individuals can gain the highest fitness by adopting a risky strategy. Individuals which accept a higher predation risk can explore more and thereby collect more resource items if they manage to survive long enough. This style, which represents a more proactive behaviour type, comes to predominate in the population because shy (reactive) types collect few resources despite suffering less predation. However, if circumstances allow for effective anti-predation learning, increased learning skills combined with high exploration tendencies become the most adaptive cognitive style. Such a fast learning and highly active cognitive style is opposed to what is commonly expected by the proactive-reaction framework, but has been found in several species (e.g. [13, 15, 35]).

When resources are present for which an investment in higher learning abilities is needed in order to exploit them, a different set of cognitive styles can be found. Under these circumstances, fast learning strategies become adaptive if lifespans are long enough to allow for handling the resources through learning. Whether individuals show high or low exploration tendencies depends both on how easily resources are found and on how severe predation pressure is.

Furthermore, we found under a large range of environmental conditions that different cognitive styles can co-exist within the same population. Due to specializing on a resource type and its interplay with optimal search pattern (exploration tendency), fast and slow styles can co-exist. Frequency-dependence of these styles may stabilize their co-existence as suggested by Boogert and colleagues [5], compare also [36]. For example, in one population some individuals can specialize on easy-to-find and easy-to-handle resources and thus exhibit a slow learning / fast exploration style, whereas other individuals can exploit resources which are hard to find and require learning abilities, thus exhibiting a fast learning / slow exploration style. Almost all other possible combinations of these two individual traits can co-exist under specific environmental circumstances in our simulations. These results can therefore help to explain why different studies find such a large variety of behaviour and cognitive styles in nature, even within the same study system and under similar environmental conditions. Furthermore, it is conceivable that in two studies either some uncontrolled variables of the environment can cause slightly different circumstances (e.g. small differences in predation pressure or in resource composition between two populations). Or, depending on the sampling regime, one of two or more co-existing cognitive styles may be captured more frequently in one study than another. When behavioural and cognitive tasks are conducted with these non-random subsets of individuals it will likely lead to different population-averages in performance.

In line with what has been suggested for individual specialisation in general [37], the co-existence of different cognitive styles may stabilize populations as microhabitats can more efficiently be occupied and within-species competition can be reduced as individuals with different styles, at least partly, exploit different resources (compare [38]). Inter-individual differences can also facilitate speciation (e.g. [39, 40]), underlining its importance for ecology and evolution in general.

In our simulations, predation strongly influences the existence of cognitive styles, as has previously been shown for behavioural syndromes (reviewed in [30]). Predation can cause the evolution of alternative styles in an otherwise similar environment. In general, predation reduces exploration tendency. But under some circumstances, this effect is not found (see also [31, 41]). For example, lifespan can be so short that individuals need to have a high exploration tendency and face the risk of predation, because otherwise they may not collect any resources at all. Or, if learning of predator avoidance is efficient enough to render the predation risk negligible, high exploration becomes more adaptive.

Furthermore, predation can also break down the co-existence by making only one strategy adaptive under given circumstances. However, predation can also cause the co-existence of cognitive styles e.g. by reducing lifespans to such an extent that investment in learning becomes less profitable, thus rendering slow-learning strategies competitive. These effects were found in a limited parameter space only, which, however, is in line with findings of predators’ effects on co-existence of interspecific competitors (reviewed in [42]).

In line with the suggestion of Sih and Del Giudice [2] we found that the influence of behaviour and cognitive traits on each other can go in both directions. The effect which these (sets of) traits have on each other’s evolution can be positive or negative (see Additional file 1: Figure S4). For example, increased exploration results in increased encounters with specific resources which allows for effective learning and thus drives the evolution of fast learning (not shown). On the other hand, increased exploration may also constrain learning because increased exploration reduces lifespan under severe predation pressure and thereby reduces opportunities to learn (compare Additional file 1: Figure S2 B).

It would be interesting to investigate how social learning may influence this pattern. For example, in group-living species, shy individuals may learn anti-predator behaviour by observing bolder or more explorative individuals coping with predator encounters. Thereby, slow explorer or shyer individuals could possibly reduce predation pressure without increasing their own predation risk by doing so. This could create an interesting interplay of the evolution of bold individual learners and shy social learners.

Of course, our simulations are based on many simplifications, which limits their transferability to natural systems. However, these simplifications allow to identify some general principles. We assumed that the trait „*L*” allows for learning in two different situations: anti-predator behaviour and handling resources. One might argue that this is an unjustified simplification as these situations represent cognitive problems from two different domains. Indeed, this could be a valid point. However, we intuitively expect that even with two independently evolving learning traits our main findings would remain qualitatively similar, i.e. that different environmental conditions can select for all combinations of exploration- and learning-styles and that these styles could in principle co-exist in the same population. Yet, certainly the parameter space under which similar strategies would be found will shift to some degree. And of course, with more evolving traits, we would likely find more cognitive styles e.g. some fast explorers which are good at anti-predator learning but slow at reducing resource handling times and vice versa.

Anyways, the assumption that learning abilities such as associative learning can be domain-general or at least underlie the performance in different cognitive tasks may not be an unjustified simplification after all. In fact, studies have shown that, at least in some taxa, animals show „general intelligence“, meaning that species, or individuals, which score high in one cognitive task also score high in tasks of other cognitive domains (reviewed e.g. in [43]). It is conceivable that mechanisms such as simple associative abilities may allow to learn in different situations and that our simulation may be realistic in this regard.

We also want to point out that, although the models presented here are based on genetic adaptation, we would expect similar outcomes if adaptive phenotypes, in our case specialized cognitive styles, would develop via developmental plasticity. Whether plastic responses are to be expected depends mostly on the timescale on which local conditions change. When environmental conditions change intermediately fast, plastic development are favoured, while under very fast or very slow changing conditions, fixed development (adjusted by genetic adaptation) dominates (e.g. [44]). Anyhow, both fixed and plastic development should usually lead to phenotypes that are adapted to local conditions. We therefore expect, as mentioned in the introduction, that the general conclusions of the present study can be transferred to systems in which differences in cognitive styles are generated by plasticity.

In this study, we regard the interplay of five aspects: exploration, learning, environmental complexity (implemented as “resource composition”), predation pressure, and maximum lifespan. We chose these aspects because they are often investigated and discussed in regard to animal personality, coping or cognitive style. However, of course, many other aspects of the environment and the species living in it are likely to influence the evolution of cognitive styles. For example, instead of handling resources, other environmental aspects may need to be learned, such as navigation through space [45], or nest-building [46]. Also, when interacting with conspecifics, cognitive styles may strongly be influenced by social learning skills. If learning is involved in interactions with other intelligent agents such as conspecifics or predators, interesting dynamics may occur in the evolution of cognitive styles. This may be a worthy field of further investigations which may help to understand the evolution of animal intelligence in general.

As a final remark we want to point out that there has been much work done, both theoretical and experimental, on the co-existence of competing species and some general conclusions may be transferable to a within-species context. Thereby, the scientific younger field of individual differences (i.e. behavioural types, coping styles, animal personality or cognitive styles) may benefit from decade-long research of interactions between species. On the other hand, no such generalisations may be possible when within-species processes such as sexual selection or kin competition are involved.

## Conclusions

The simulations show that different environmental conditions can select for different cognitive styles. Under a wide range of parameter settings, individuals of the same population may adopt different cognitive styles that co-exist in an often frequency-dependent manner. Showing how different cognitive styles may lead to similar fitness even within the same environment can help to explain the variety of styles described in previous studies and why different, sometimes contradicting, results have been found. We were also able to show how behaviour traits may influence the evolution of cognitive traits and vice versa, illustrating the coevolutionary dynamics leading to cognitive styles.

## Additional file

**Additional file 1: Figure S1.** Predation prevents the emergence of fast exploration strategy. **Figure S2.** Predation hinders co-existence. **Figure S3.** Predation can also induce co-existence. **Figure S4.** Influence of behavioural traits on cognitive traits and vice versa.

## Data Availability

Code of simulation and data is available in Dryad (10.5061/dryad.zw3r2284m).

## Abbreviations

*C*: Cumulative exploration tendency
*D*_*Ri*_: Detectability = how difficult resource i is to find
*E*: Exploration tendency
*F*: Reproductive success
*H*_*Ri*_: Time to handle resource type i
*L*: Learning ability = learning speed
*N*_*Generations*_: Number of generations
*N*_*Individuals*_: Population size
*N*_*Sites*_: Number of sites in the environment
*N*_*Steps*_: Number of time steps per day
*P*_*p*_: Baseline probability of encountering predators
*P*_*Ri*_: Proportion of sites filled with resource *R*_*i*_.
*q*: Mutation probability
*R*_*i*_: Names for resource i
*S*: Selectiveness
*ß*: Parameter defining the general speed of predation-learning
*T*: Number of days = season length = lifespan
*V*_*Ri*_: Value of resource i
*V*_*Total*_: Total value of resources collected by an individual
*α*: Cost coefficient that specifies the cost of learning
*λ*_*P*_: Lethality of predators = likelihood of dying when encountering a predator
