# Supplementary material for: Modelling the evolution of cognitive styles
Source: BMC Evol Biol. 2019 Dec 27;19:234. doi: 10.1186/s12862-019-1565-2 (PMC6935132; doi:10.1186/s12862-019-1565-2)
Supplement: Supplementary file 1 — Additional file 1: Figure S1. Predation prevents the emergence of fast exploration strategy. Figure S2. Predation hinders co-existence. Figure S3. Predation can also induce co-existence. Figure S4. Influence of behavioural traits on cognitive traits and vice versa. [file 12862_2019_1565_MOESM1_ESM.docx]

**Supplementary Material**

EVOB-D-19-00211

Modelling the evolution of cognitive styles

Jannis Liedtke; Lutz Fromhage

BMC Evolutionary Biology

**Figure S1**


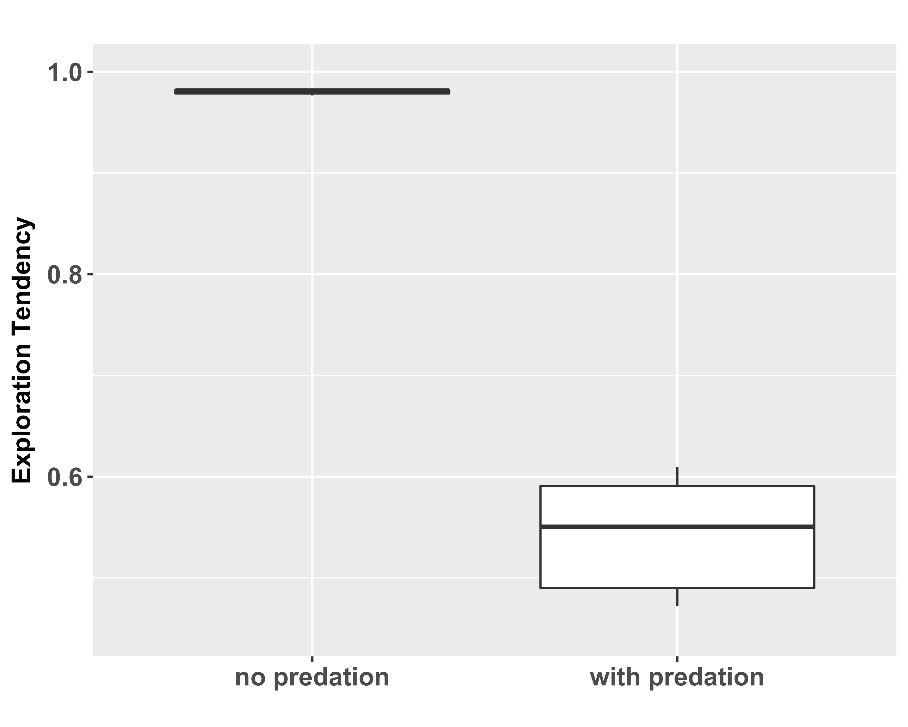


**Figure S1: Predation prevents the emergence of fast exploration strategy**

Each boxplot is based on 10 replicate simulations with *N* = 1000 and *G* = 500; *D_R1_* = 0.9 and *D_R2_* = 0.9; *T* = 200. The only difference in parameter settings is predation.

Figure S2


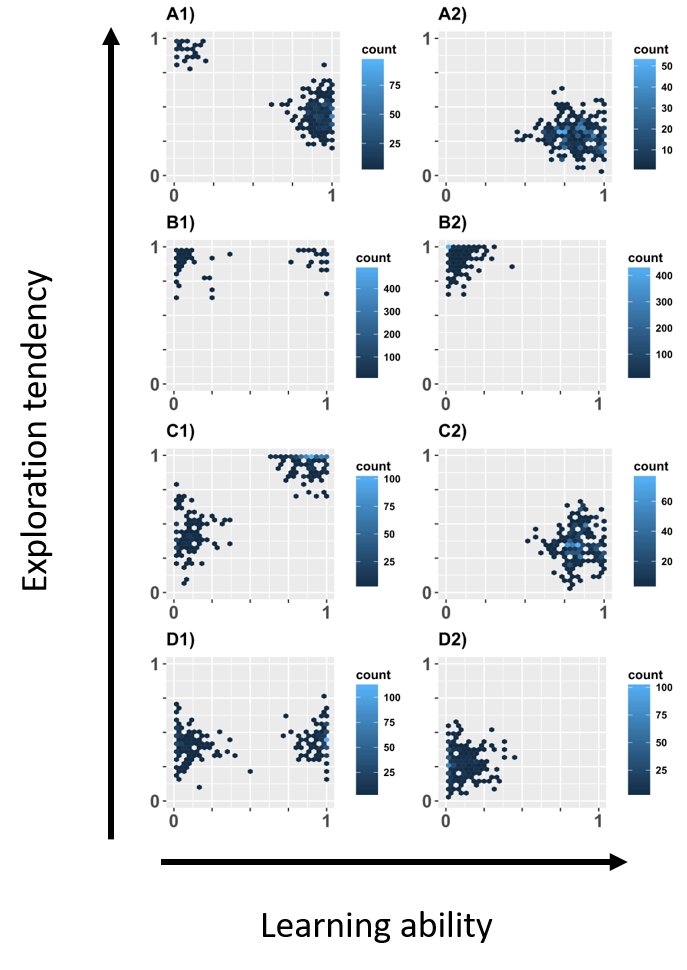


**Fig. S2: Predation hinders co-existence**

Each panel shows the result of one simulation as an example from 10 replicate runs. In each figure pair (e.g. A1 and A2 etc.) left hand panels show the results of simulations without predation. Corresponding right hand panels show simulations with otherwise identical setting but with predation. Conditions between different pairs (i.e. A, B, C, and D) vary in resource detectability (*D_Ri_*) and season length (*T*). Predation can hinder co-existence of cognitive styles in multiple ways. For example, by making fast-exploration and slow-learning strategies (A) or fast-exploring and fast-learning (B) less beneficial. In C) a slow learning strategy becomes less adaptive, as slow learners are not able to learn sufficiently to reduce lethality of predators. In D) even fast learning is not enough to reduce predation sufficiently and fast-learning styles are prevented. Settings: A: *D_R1_* = 0.9 and *D_R2_* = 0.0; *T* = 150; B: *D_R1_* = 0.9 and *D_R2_* = 0.9; *T* = 20; C) *D_R1_* = 0.0 and *D_R2_* = 0.9; *T* = 200; D) *D_R1_* = 0.0 and *D_R2_* = 0.0; *T*= 45.

Figure S3


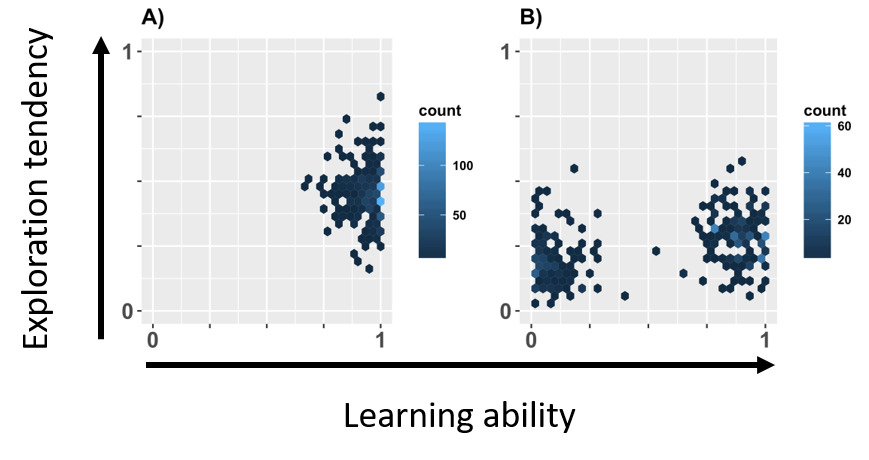


**Figure S3: Predation can also induce co-existence**

Results for simulations A) without predation and B) with predation. All other parameter settings were identical. Both panels show the result of one simulation as an example from 10 replicate runs. All replicate runs produced qualitatively similar results, with one exception: in one out of ten runs in B), the co-existence collapsed due to the extinction of the fast learning strategy, which was likely caused by a combination of stochastic events and high predation pressure. Settings: *D_R1_* = 0.0 and *D_R2_* = 0.0; *T* = 110.

Figure S4


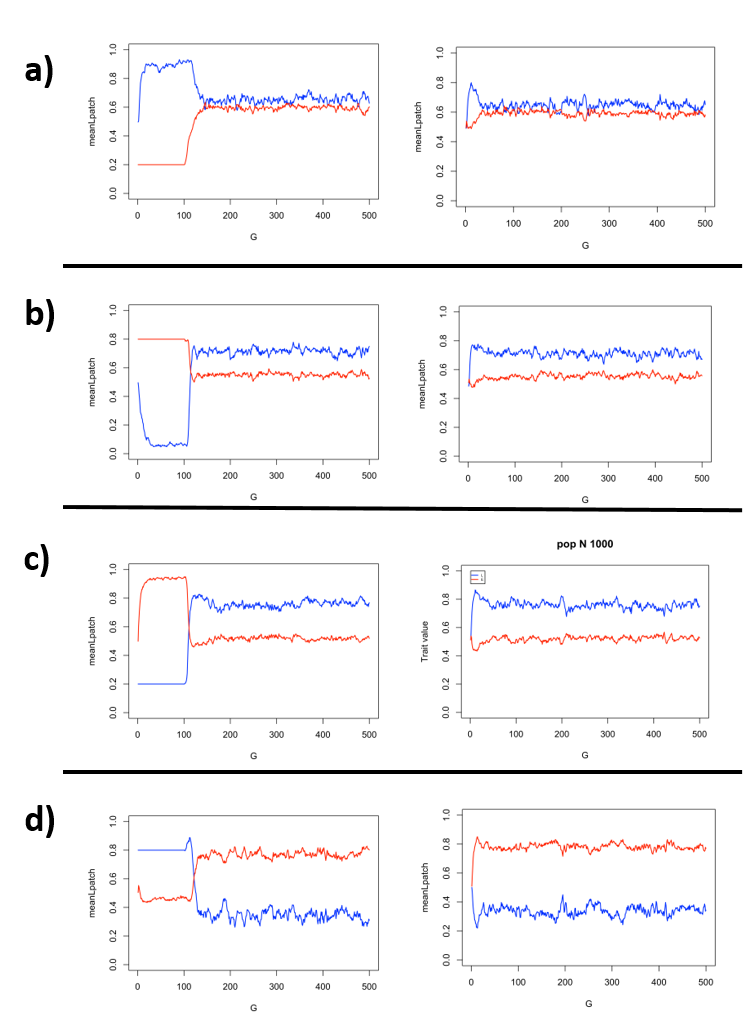


**Figure S4: Influence of behavioural traits on cognitive traits and vice versa**

Each panel shows the result of one simulation as an example from 10 replicate runs. All replicates produced qualitatively similar results. Panels on left-hand side show results of simulations in which the value for one trait (exploration tendency (*E*) in red; learning speed (*L*) in blue) was fixed (for *E* in a and b; for *L* in c and d) for the first 100 generations to either a low trait value (a and c = 0.2) or a high value (b and d = 0.8). After 100 generations both traits were equally subject to mutation and could evolve to any value between 0 and 1. Panels on the right-hand side show results of simulations with identical settings as on the left-hand side but both traits could freely mutate from the beginning. Parameter settings were: *N*=1000; *G*=500; *λ*=0.1; *P*=0.025 for all simulations and season length (*T*) was in a=80; in b=90; in c=100 and in d=60.
